# The relationship between changes in health behaviour and initiation of lipid-lowering and antihypertensive medications in individuals at high risk of ischaemic heart disease

**DOI:** 10.1186/1471-2458-12-626

**Published:** 2012-08-08

**Authors:** Nana Folmann Hempler, Allan Krasnik, Charlotta Pisinger, Torben Jørgensen

**Affiliations:** 1 Center for Healthy Aging, Section for Health Services Research, Department of Public Health, University of Copenhagen, 1014 Copenhagen K, Denmark; 2 Steno Health Promotion Center, Steno Diabetes Center, 2820, Gentofte, Denmark; 3 Research Centre for Prevention and Health, Capital Region, Glostrup University Hospital, 2600, Glostrup, Denmark; 4 Faculty of Health Sciences, University of Copenhagen, 1014, Copenhagen K, Denmark

## Abstract

**Background:**

It has been hypothesised that health conscious individuals tend to take better care of themselves by greater adherence to preventive medications. We examined, whether long-term changes in dietary habits and physical activity were associated with initiation of lipid-lowering and antihypertensive medications.

**Methods:**

The study population consisted of two subsamples from the population-based cohort Inter99 study (1999-2006) in Copenhagen, Denmark: one with systolic blood pressure > 140 mmHg (N = 557) and one with total cholesterol > 7 mmol/L (N = 314). At a health examination, individuals completed a questionnaire about health behaviour and had their blood pressure and cholesterol measured at baseline and after five years. Data on medications were obtained through linkage to the Registry of Medical Product Statistics.

**Results:**

Positive changes in physical activity (odds ratio =3.50; 95% CI 1.23-7.54) and in dietary habits (odds ratio = 2.08; 95% CI 1.03-4.21) were associated with an increased initiation of lipid-lowering medications. With respect to antihypertensives, no association was observed in terms of physical activity, but for diet, a positive trend in terms of initiation was observed among those with positive changes in dietary habits (odds ratio = 1.58; 95% CI 0.96-2.59).

**Conclusion:**

Generally, we observed health conscious behaviour in terms of increased initiation of preventive medications among those who reported positive changes in health behaviour. This study therefore suggests that more attention should be given to identifying individuals and groups, who are less health conscious and thereby less likely to engage in either preventive medications or changes in health behaviours.

## Background

Non-pharmacological treatment, in terms of physical activity, dietary habits and smoking cessation, has been shown to have a beneficial effect on biological cardiovascular disease (CVD) risk factors. Physical activity is known to modify well-known CVD risk factors, such as, hypertension, dyslipidemia, abdominal and general obesity [1-5]. Also, individual dietary interventions in primary prevention have shown moderate effects in relation to cardiovascular risk factors, such as, blood pressure, total cholesterol, LDL cholesterol stemming from dietary changes in terms of lower salt and fat, higher fibre and fruit and vegetable consumption [6-9]. Furthermore, studies have shown that quitting smoking reduces the risk of acute myocardial infarction [10,11].

Regarding pharmacological treatment, there have been well-reasoned and balanced recommendations for the use of statins for individuals, who have an elevated risk of cardiovascular events [12-14]. Furthermore, blood-pressure lowering drugs reduce the risk of coronary heart disease (CHD) events and stroke, also in individuals with no previous history of cardiovascular disease [15]. There is also evidence that users of medications for hypertension and hypercholesterolemia may benefit from health behaviour changes such as improved diet, quitting smoking and exercise, particularly in the setting of primary prevention [16]. Danish national guidelines, from 2007, recommended that the general practitioner should motivate people at high risk of CVD (10-year absolute risk of CVD death ≥ 5%), and with no pre-existing CVD, to pursue health behaviour changes within 3 months, before considering pharmacological treatment [17]. Only in terms of a very high risk (10-year absolute risk of CVD death ≥ 10%), the general practitioner should not hesitate to initiate pharmacological treatment.

Several observational studies have reported that individuals who initiate and adhere to preventive therapy such as statin use are likely to be more health conscious, the so-called healthy adherer effect [18-21]. ‘Healthy adherer patients’ are more likely to see their doctors on a regular basis, exercise, have healthier diets, stop smoking, adhere to treatment and avoid risky behaviours.

In the present study, we investigated a possible healthy adherer effect among individuals with an elevated risk of ischaemic heart disease (IHD) in terms of high blood pressure and high total cholesterol level. We hypothesised that individuals who were able to change their health behaviours, also were more likely to initiate treatment with lipid-lowering medications and blood pressure-lowering medications, rather than substituting medications with behavioural change. To explore this, we examined whether long-term changes in dietary habits and physical activity were associated with initiation of preventive medications. We conducted the study by using two subsamples from the population-based cohort from the Inter99 study.

## Methods

### Study population

The study population comprised of two subsamples of participants from the Inter99 study, which is a population-based randomised controlled trial, investigating the effect of non-pharmacological intervention on cardiovascular disease [22]. The study was performed at the Research Centre for Prevention and Health, Denmark, approved by the Copenhagen County Ethical Committee (KA 98155) and registered in the Clinical Trials.gov (NCT00289237).

The Inter99 study was initiated in 1999 with a subsequent five-year follow-up. The study population comprised all individuals born in 1939-40, 1944-45, 1949-50, 1554-55, 1959-60, 1964-65, 1969-70 and living in 11  municipalities in the southern part of Copenhagen County, and who were identified in the Civil Registration System. The initial entire study population consisted of 61,301 participants and was pre-randomised into three groups: a high intervention group (Group A, N = 11,708), a low intensity intervention group (Group B, N = 1,308) and a control group (N = 48,285). The inclusion criteria for the study population in the present article were: (1) randomised to group A; (2) information on dietary habits and physical activity at baseline and five-year follow-up; (3) high blood pressure (>140 mmHg) (N = 557) or high total cholesterol (>7 mmol/L) (N = 314) measured at baseline.

### The intervention

The baseline examination took place between November 1999 and January 2001. The participants were invited for an initial health examination and their risk of ischemic heart disease was assessed at baseline. Using the Copenhagen Risk Score, the 10-year absolute risk of IHD was calculated (mean of sex, age, heredity, former IHD, diabetes, height, weight, smoking habits, cholesterol and blood pressure) by the computer program “PRECARD*®*” [23]. At baseline, 52% of the initially invited individuals in group A, corresponding to 6,091 men and women, participated in the baseline examination. At baseline, all participants at risk received a lifestyle consultation encouraging them to healthy behaviour (focusing on smoking, diet, physical activity and reduction in alcohol consumption). Participants in group A were offered group-based counselling in relation to smoking cessation and diet/physical activity, but participation was relatively low at baseline, and adherence was poor. All participants were re-invited after one-, three- and five years. At five-year follow-up, the health examination programme was repeated. Furthermore, all participants with high blood pressure, cholesterol or glucose received a print-out of the results for their general practitioner and were recommended to contact him/her.

### Assessment of dependent variables

The Registry of Medical Product Statistics contains information on all outpatient prescriptions in Denmark using the Anatomical Therapeutic Chemical Classification System (ATC codes). By linking survey data with registry data using Danish unique personal identification numbers, we identified data on prescriptions related to lipid-lowering (code C10A and C10B) and antihypertensive medications (code C02, C03, C7, C8, C09). We used the 2007 version of the ATC/DDD classification. Initiation was defined as a least one redeemed prescription at a pharmacy in the period from baseline participation to the end of the 5-year follow-up. Participants who reported using the abovementioned medications at baseline were excluded from the analyses.

### Health behaviour

Information on physical activity and dietary habits was obtained by questionnaire. Total physical activity was calculated on the basis of two questions, on commuting physical activity and leisure time physical activity [24]. The variable was calculated by summing responses to commuting physical activity (converted into minutes per week using a five day working week) and leisure time physical activity (converted into minutes per week). Physical activity was defined as: decreased/unchanged level and increased level. Change in dietary intake (nine-class variable) was measured by a dietary quality score (intake of fish, vegetables, fruit and fat) using a self-administrated 52-item food questionnaire (reference period: the last week). The questionnaire had been validated using a 28-day diet history and biomarkers as reference methods [25]. The nine-class variable was dichotomised into two groups: healthier and unchanged/more unhealthy. None of the participants in this study population received a maximum score of 9 (the healthiest) at baseline. Particularly, the groups that were more unhealthy or had decreased values at follow-up were relatively small, and therefore both health behaviour variables were dichotomised, due to small sample size.

### Other covariates

Information on age and sex was taken from the Civil Registration System. From the Integrated Database for Labour Market Research at Statistics Denmark, information on educational attainment at baseline was obtained. Educational attainment was dichotomised into “high” (at least secondary education) and “low” (primary education only). Education is likely to be a good proxy for income level, as expenditures for medication require co-payments, while visits to the general practitioners are free of charge in Denmark. Information on contacts with general practitioners was obtained by linking to the National Health Insurance Service Registry. At the health examination, participants provided fasting blood samples for assessment of total cholesterol. Total cholesterol was measured by enzymatic procedures (Boeringer Mannhein, Germany). Blood pressure (BP) was measured twice with a mercury sphygmomanometer after 5 min of rest in a lying position. Height was measured without shoes to the nearest cm, weight was measured without shoes and overcoat to the nearest 0.1 kg and body mass index (BMI) was calculated. Risk of ischemic heart disease was assessed by using the “Copenhagen Risk Score” [23]. Smoking habits at baseline were assessed by answers to self-report questions at baseline.

### Statistical analysis

Data were analysed using SAS statistical software, version 9.2 (SAS Institute., Cary, NC, USA).

The associations between initiation of preventive medications and changes in health behaviour were explored by logistic regression models. We excluded those with diabetes, as guidelines for patients with diabetes differ from those without diabetes. Regarding analyses for physical activity, those who reported low physical activity at baseline due to illness or handicap were excluded (N = 14). We examined three models of the relationship between change in health behaviour or initiation of lipid-lowering and antihypertensive medications. In model 1, we included age, sex and baseline values for physical activity (in analyses of change in physical activity) or dietary habits (in analyses of change in dietary habits). In model 2, we included covariates of health behaviour and educational level, and in model 3, we adjusted for risk score, BMI and in models regarding lipid-lowering medications, we adjusted for cholesterol at baseline, and in models regarding antihypertensives were adjusted for blood pressure level. As a model control, we carried out Hosmer and Lemeshow goodness-of-fit tests for the logistic regression models. Linearity of age, cholesterol and blood pressure were tested, and when needed, we squared the number, or raised it to the third power. We tested plausible interaction terms between the primary explanatory variable and sex and education.

## Results

Of the baseline participants in this study, about 62% in both subsamples returned for a five-year follow-up during 2004-2006 (Table1). Compared with responders, non-responders in the sample with high blood pressure comprised more women (47.0%), daily smokers (36.3%), diabetics (4.2%), persons with primary education only (29.5%) and individuals with alcohol consumptions above recommended levels (25.6%). Non-respondents in the sample with high total cholesterol comprised more women (51.5%), individuals with more than primary education (40.3%), daily smokers (56.6%) and 30-35 year-olds (7.1%).

**Table 1:** Socio-demographic, health behaviour and biological measurements in the study population

	**Blood pressure > 140 mmhg**	**Total cholesterol > 7 mmol/L**
	**(n = 557)**	**(n = 314)**
**Sex**		
Men	320 (57.5%)	187 (59.6%)
Women	237 (42.5%)	127 (40.4%)
**Age group (years)**		
30-35	17 (3.1%)	13 (4.1%)
40-55	266 (47.7%)	166 (52.9%)
55-60	274 (49.2%)	135 (43.0%)
**Educational level**		
More than primary education	421 (75.6%)	66 (21.0%)
Primary education only	131 (23.5%)	248 (79.0%)
Missing	5 (0.9%)	
**Diabetes**		
Positive test	9 (1.6%)	3 (1.0%)
**Smoking status**		
Daily	136 (24.4%)	107 (34.1%)
Occasional	19 (3.4%)	11 (3.5%)
Ex-smoker	172 (30.9%)	78 (24.8%)
Never smoked	228 (40.9%)	118 (37.6%)
Missing	2 (0.4%)	
**Alcohol**		
Abstinent	50 (9.0%)	19 (6.0%)
Within recommendations	385 (69.1%)	227 (72.3%)
Above recommendations	103 (18.5%)	64 (20.4)
Missing	19 (3.4%)	4 (1.3%)
**Physical activity (minutes per week)**		
0-112.5	59 (10.6%)	31 (9.9%)
142.5-225	122 (21.9%)	79 (25.2%)
255-420	263 (497.2%)	145 (46.2%)
450-720	74 (13.3%)	31 (9.9%)
Missing	39 (7.0%)	28 (8.9%)
**Dietary habits**		
Unhealthy dietary habit	88 (15.8%)	44 (14.0%)
Average dietary habits	371 (66.7%)	215 (68.5%)
Healthy dietary habits	83 (14.9%)	42 (13.4%)
Missing	15 (2.7%)	13 (4.1%)
**Body mass index, kg/m**^**2**^		
Mean	28.4	27.0
**Systolic blood presure, mm/Hg**^**a**^		
Mean	155.8	
**Cholesterol**^**b**^		
Mean		7.7
**At least one contact with GP**		
Within 1 year after baseline	99.6%	100%

a: Participants who report current use of antihypertensives (n = 128) were excluded.

b: No participants reported use of lipid-lowering medications within the last few weeks.

**Figure 1:**
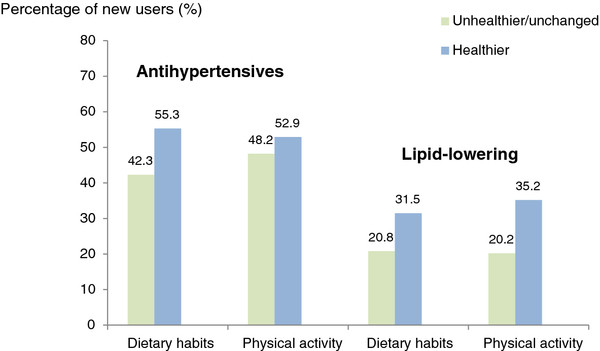
The relationship between changes in health behaviour and initiation of antihypertensives and lipid-lowering medications at 5-year follow-up.

For individuals who reported an increase in physical activity at five-year follow-up, we observed a higher percentage of new users of lipid-lowering medications (p = 0.0117) and antihypertensives (p = 0.4477) compared with those with a decreased or unchanged level (Figure1). With respect to diet, we also observed a higher percentage of new users of lipid-lowering medications (p = 0.0379) and antihypertensives (p = 0.0092) among those with positive health behaviour changes, compared with those with unchanged/unhealthier diet (Figure1).

### Initiation of antihypertensive medications

In multivariate analyses, positive changes in physical activity were not associated with initiation of antihypertensives (Table2), which was also the case in the fully adjusted model (model 3). Regarding dietary habits, positive changes were significantly associated with increased initiation of antihypertensives (model 1 and model 2). When we included risk score, blood pressure level and BMI in model 3, the association between positive changes and initiation of antihypertensives remained positive, but was no longer statistically significant (model 3).

**Table 2:** **Effect of five-year change in health behaviour on initiation of antihypertensive medications**^**a**^

	**N**	**Initiation of medication**	**Initiation of medication**	**Initiation of medication**
		**Model 1 OR (95% CI)**	**Model 2 OR (95% CI)**	**Model 3 OR (95% CI)**
**Physical activity**				
Unchanged/decrease	280	1	1	1
Increase	87	1.00 (0.54-1.84)	1.03 (0.55-1.93)	1.09 (0.55-2.15)
**Dietary habits**				
Unchanged/more unhealthy	189	1	1	1
Healthier	219	**1.59 (1.03-1.44)**	**1.64 (1.03-2.60)**	1.58 (0.96-2.59)

Model 1: Age, sex, baseline values for physical activity/dietary habits.

Model 2: +Education, health behaviour (smoking status, alcohol, diet/physical activity).

Model 3: +Risk score, blood pressure level, BMI.

a: Participants who report use of antihypertensives (baseline, n = 128) were excluded.

### Initiation of lipid-lowering medications

With respect to physical activity, positive changes were significantly associated with an increased initiation of lipid-lowering medications (Table3, model 1). The association remained significant when the models were adjusted for educational level, health behaviour, risk score, cholesterol and BMI. Regarding dietary habits, initiation of lipid-lowering medications was more frequent in the group that reported positive changes, compared with those with unchanged/more unhealthy dietary habits, but estimates were not significant (model 1). After adjusting for risk score, cholesterol level and BMI, the association was statistically significant (model 3).

**Table 3:** Effect of five-year change in health behaviour on initiation of lipid-lowering medications

	**N**	**Initiation of medication**	**Initiation of medications**	**Initiation of medication**
		**Model 1 OR (95% CI)**	**Model 2 OR (95% CI)**	**Model 3 OR (95% CI)**
**Physical activity**				
Unchanged/decrease	193	1	1	1
Increase	71	**2.96 (1.34-6.53)**	**3.01 (1.31-6.90)**	**3.05 (1.23-7.54)**
**Dietary habits**				
Unchanged/more unhealthy	149	1	1	1
Healthier	143	1.61 (0.90-2.86)	1.85 (0.97-3.53)	**2.08 (1.03-4.21)**

Model 1: Age, sex, baseline values for physical activity/dietary habits.

Model 2: +Education, health behaviour (smoking status, alcohol, diet/physical activity).

Model 3: +Risk score, cholesterol level, BMI.

### Additional analyses

We repeated analyses for model 3 and adjusted for participation/nonparticipation in a diet and exercise group-based counseling programme. A similar pattern was found for the four outcomes (data not shown). We also compared initiation of medications between the participants and the drop-outs, and adjusted for all baseline covariates. Regarding initiation of lipid-lowering medications (odds ratio = 1.02; 95% CI 0.56-1.31) and antihypertensives (odds ratio = 1.01; 95% CI 0.69-1.48), no notable differences were observed between the groups.

## Discussion

The main finding of this study was that positive changes in dietary habits were associated with increased initiation of lipid-lowering and antihypertensive medications. Furthermore, participants, who reported an increase in terms of physical activity were more likely to initiate treatment with lipid-lowering medications; however, in terms of antihypertensives, no association was observed. Some associations may be non-significant due to type 2 errors resulting from the relatively small sample size. Our hypothesis that people who were able to change their health behaviour also would tend to take better care of themselves, by greater adherence to preventive medications, was therefore supported to some extent. The purpose of this study was not to assess whether patients with increased blood pressure or total cholesterol ought to initiate treatment with preventive medications and/or change in health behaviour, but to shed light on a possible health conscious behaviour and thereby also lack of health consciousness in other individuals.

A study by Brookhart et al. reported that patients who adhered to statin therapy underwent a variety of cancer screening tests, received influenza and pneumococcal vaccinations, compared with those who were less adherent [26]. In another study by Majumdar et al., focusing on patients admitted to hospital with community acquired pneumonia, statin users were more likely to be former smokers and have up-to-date immunizations for pneumococcus and influenza [18]. Furthermore, Dormuth et al. reported that patients with good adherence to therapy with statins were less likely to be involved in motor vehicle or work place accidents requiring medical attention than less adherent patients [20]. Although the studies differ in terms of design, outcome measure and definition of adherence, they all suggest a healthy adherer or healthy user effect.

Medical treatment with preventive medications is likely to be determined by system-, provider, as well as patient-level factors, and the factors also interact [27]. In this study, it might be speculated that it was the most health conscious participants who consulted their general practitioner regarding their increased risk of IHD. The most health conscious individuals may be more open-minded regarding preventive medications and may even demand such treatment. Nevertheless, health consciousness in itself does not lead to the prescribing of medications. In Denmark, primary prevention of cardiovascular disease is often handled by the general practitioners. Almost all participants in our study visited their general practitioner within one year after baseline (Table1), but it remains unknown, how many actually consulted their general practitioners due to their increased risk of IHD and/or motivation for health behaviour change, as the Health Insurance Registry does not provide information about diagnoses related to contacts.

On the other hand, participation in a non-pharmacological intervention (as with the Inter99 study) might have affected the general practitioners’ decisions regarding treatment, in terms of a hesitation towards initiating treatment. Generally, the prescribing of preventive medications has been much debated, also among general practitioners. A recent Cochrane review, reported a stronger effect of statins on overall mortality than observed in previous meta-analyses [14], but at the same time the authors did not support the widespread use of statins in individuals without a previous history of heart disease, due to high costs and risks associated with side effects [28]. Another fact is that Danish national guidelines from 1998 and 2002 were less likely to recommend pharmacological treatment as primary treatment for IHD, as was the case in the 2007 guideline. Also, general practitioners were very reluctant to initiate treatment with statins, as the price was very high and regulations on subsidies were very complicated in the first years of the study. This is likely to explain the relatively low prevalence of users.

Generally, participants with unchanged/negative changes in health behaviour were less likely to initiate treatment with preventive medications, but this was not the case for antihypertensives and physical activity, where no healthy adherer affect was observed. One explanation for this finding might be that physicians have a tendency to prescribe medications to patients, who are most likely to benefit from them [27], for example as a result of unhealthy behaviours or poorer health status. However, we tried to take this into consideration, by excluding those who reported a decrease in physical activity due to illness/handicap and by adjusting for blood pressure/cholesterol and risk score. Differences in health status did appear to have some effect, as some associations were strengthened by the inclusion of the above mentioned variables.

The generalisability of our study should be made carefully, as our study population is relatively young, received lifestyle intervention and were referred to their general practitioners. The strengths of this study are the follow-up design and the fact that we were able to link data to the Danish National Prescription Registry of high validity [29]. Our study also has limitations. The relatively low participation rate at baseline might cause some selection bias, because group A had lower BMI than the general population (group C), but poorer lifestyle [22]. Nevertheless, the intervention groups have been found to be representative for the general population (group c) with regard to former admissions for all causes, IHD, CVD and diabetes [22]. Another thing to keep in mind is the selection in drop-out at five-year follow-up. It is likely that it was the most health conscious individuals who returned at follow-up. However, we consider this less relevant, as we investigate associations for participants with complete data. Furthermore, we cannot exclude the risk of residual confounding due to broad categories of dietary habits and physical activity. A weakness is also that health behaviour data are self-report, which involves a risk of bias and misclassification. It cannot be ruled out that some participants may have reported too positive changes in health behaviour; however, this would lead to underestimated associations between changes in health behaviour and initiation of medications. Previous cardiovascular events might also cause a selection and therefore affect the findings; but, due to the relatively young population, and the fact that we excluded those with existing use of medications and diabetes, we consider this less relevant. We might have underestimated the associations, as participants who were the healthiest at baseline may have difficulties in increasing their scores; the so-called ceiling effect. Finally, we did not consider subgroups of medications, combinations of different medications or the fact that comorbidity, such as with migraines, might require antihypertensives.

## Conclusion

The study partially supports the hypothesis of health conscious behaviour in terms of increased initiation of preventive medications among those who experienced positive changes in health behaviour. This fact implies that studies that examine the effect of lipid-lowering and antihypertensive medications might consider the healthy adherer effect in terms of a potential bias. From a public health perspective, our study suggest that more attention should be given to identifying individuals and groups, who are less health conscious and thereby less likely to engage in either preventive medication or changes in health behaviour. Further research is needed to understand the complex interactions between healthy behaviour and preventive medication and the role of individual perceptions of health, health behaviour and medication.

## Competing interests

The authors declare they have no competing interests.

## Authors contribution

NFH conceived the study, conducted the analysis of the data and had primary responsibility for drafting the manuscript. All authors contributed to the design of the study and participated in the interpretation of results and critically reviewed the manuscript. All authors read and approved the final manuscript.

## Pre-publication history

The pre-publication history for this paper can be accessed here:

http://www.biomedcentral.com/1471-2458/12/626/prepub
